# Exposure to Major Vector-Borne Diseases in Dogs Subjected to Different Preventative Regimens in Endemic Areas of Italy

**DOI:** 10.3390/pathogens10050507

**Published:** 2021-04-23

**Authors:** Mariasole Colombo, Simone Morelli, Giulia Simonato, Angela Di Cesare, Fabrizia Veronesi, Antonio Frangipane di Regalbono, Laura Grassi, Ilaria Russi, Pietro Giorgio Tiscar, Giulia Morganti, Jasmine Hattab, Valeria Rizzo, Donato Traversa

**Affiliations:** 1 Faculty of Veterinary Medicine, University of Teramo, 64100 Teramo, Italy; mcolombo@unite.it (M.C.); smorelli@unite.it (S.M.); adicesare@unite.it (A.D.C.); ilaria.russi.03@gmail.com (I.R.); pgtiscar@unite.it (P.G.T.); jhattab@unite.it (J.H.); valyrizzo@yahoo.com (V.R.); 2 Department of Animal Medicine, Production and Health, University of Padua, 35020 Legnaro, Italy; giulia.simonato@unipd.it (G.S.); antonio.frangipane@unipd.it (A.F.d.R.); laura.grassi.2@phd.unipd.it (L.G.); 3 Department of Veterinary Medicine, University of Perugia, 06126 Perugia, Italy; fabrizia.veronesi@unipg.it (F.V.); morganti.giulia@alice.it (G.M.)

**Keywords:** canine vector-borne diseases, dogs, ticks, sandflies, fleas, ectoparasiticides

## Abstract

Vector-borne diseases (VBDs) are globally widespread arthropod-transmitted diseases with a significant impact on animal and human health. Many drivers have recently spurred the geographic spread of VBDs in dogs. This study has evaluated the exposure to most important VBDs in dogs under different preventative treatments in different regions of Italy, i.e., Veneto, Friuli Venezia-Giulia, Umbria, Giglio Island (Tuscany), Abruzzo and Latium. Serological analyses were performed to detect antibodies against *Leishmania infantum*, *Babesia canis*, *Anaplasma phagocytophilum*/*Anaplasma platys*, *Ehrlichia canis/Ehrlichia ewingii*, *Borrelia burgdorferi, Rickettsia conorii* and the circulating antigen of *Dirofilaria immitis.* Dogs were categorized according to the treatment schedule usually received, and the association between seropositivity and possible risk factors was statistically evaluated. Overall, 124/242 (51.2%) dogs tested positive for at least one pathogen, while 34 (14.0%) were exposed to two or more pathogens. The most detected seropositivity was against *R. conorii*, followed by *Anaplasma* spp., *L. infantum*, *B. canis*, and the other pathogens under study. Significant statistical associations were found according to geographical provenance, history of tick infestation, lifestyle and inadequate prophylactic treatments. Random/irregular treatments have been identified as a clear risk factor. These results show that adequate prophylactic treatment protocols are overlooked by dog owners, despite the availability of several effective products, with possible implications in veterinary medicine and on public health.

## 1. Introduction

Canine vector-borne diseases (CVBDs) are caused by several pathogens (parasites, bacteria and viruses) transmitted by ectoparasites, namely ticks, fleas, mosquitoes and sand flies [1,2,3]. These pathogens represent a threat for human and animal health throughout continents [4,5,6,7,8,9,10].

In Europe, heartworm disease caused by the mosquito-borne nematode *Dirofilaria immitis*, leishmaniosis and babesiosis by the protozoans *Leishmania infantum* and *Babesia* spp. transmitted by sandflies and ticks, respectively, are the most important parasitic vector-borne diseases (VBDs) affecting dogs [11,12,13]. Bacterial tick-borne diseases (TBDs) also play an important role in canine medicine, as *Ehrlichia* spp., *Anaplasma* spp., *Borrelia* spp. and *Rickettsia conorii* are the most common throughout Europe and elsewhere [14,15,16,17]. Most of the abovementioned pathogens have a zoonotic potential and represent a threat for human health [2,18]. 

Several factors, including climate change and global warming, may promote the biology and spreading of vectors, while globalization, increased travelling of companion animals with their owners, relocation and the rapid growth of human and canine population have caused a geographic expansion of CVBDs into both endemic and formerly unaffected regions [1,19,20,21]. 

The Mediterranean basin is a suitable environment for the circulation of VBDs in domestic animals; thus, monitoring local canine populations and updated epidemiological data are crucial because available information is often limited to specific countries or to selected pathogens [15,22,23,24]. Recent studies have demonstrated that several zoonotic VBDs are shared between dog and cat populations throughout the Mediterranean basin, inevitably increasing the chances of spreading among pet populations and transmission to people [25,26,27]. 

The most effective strategy to minimize the risk of VBDs in pets and people in Europe must aim to reduce the exposure of animals to vectors using efficacious administrations of ectoparasiticides and anti-feeding products [28]. Very few preventative methods are available as alternatives. For instance, vaccines are marketed in some countries only for selected VBDs, i.e., leishmaniosis, babesiosis and borreliosis [28]. However, these vaccines are used only in a few cases on relatively large numbers; thus, to date, the control of CVBDs mainly relies on chemicals with insecticide/acaricide/antifeeding activity [28]. Several products are available on the market for the reliable protection of dogs and indirectly, people, from VBDs. Nevertheless, a lack of adherence to veterinary recommendations or guidelines, in terms of the choice of molecules and dosing interval, has a negative impact on control programs. A reduced compliance of dog owners could be caused by several reasons, e.g., limited financial resources, little knowledge of the products and indications and erroneous perceptions of the importance of preventative treatments [29,30]. 

The present study aimed to investigate the exposure to primary VBDs in privately owned dogs living in different regions of Italy endemic for CVBDs, to (i) evaluate the impact of different preventative regimens in their distribution and to (ii) update national epidemiological data. Risk factors associated with the seropositivity to one or more pathogens were also assessed.

## 2. Results

More than half of the study dogs, i.e., 124 (51.2%), were positive for at least one pathogen and, of them, 117 were positive by at least one TBD: 98 (40.5%) dogs were positive for *R. conorii*, 25 (10.3%) for *B. canis*, 22 (9.1%) for *Anaplasma* spp., 11 (4.5%) for *L. infantum*, 4 (1.7%) for *B. burgdorferi*, 1 (0.4%) for *Ehrlichia* spp. and 4 (1.7%) had *D. immitis* circulating antigens. Moreover, 90 (37.2%) dogs were positive for only one pathogen, while 34 (14.0%) were seropositive for two or more pathogens. Detailed results according to each single Site are listed in Table 1. 

Ticks and fleas were detected during the sampling procedures in 3 (1.2%) and 30 (12.4%) dogs, respectively, while the owners referred previous infections by ticks and fleas in 128 (52.9%) and 73 (30.2%) dogs, respectively (Table 2).

One hundred and fifty-seven (64%) dogs were permanently housed outdoors, while the remaining 85 (35.1%) dogs lived mostly indoors.

Overall, 61 dogs (25.2%) were subjected to regular treatments all-year-round, 48 (19.8%) received ectoparasiticides/anti-feeding products from the spring to autumn, 19 dogs (7.9%) only in the summer and 114 (47.1%) received random/irregular treatments. Among the latter category, no detailed data were available for 17 dogs (7.0%), because the owners were unable to specify the used formulations. In total, 134 (55.4%) and 153 dogs (63.2%) did not receive adequate prophylaxis for ticks/fleas and sandflies, respectively. Detailed information on molecules and treatment schedules received by dogs and positivity to the VBDs detected are listed in Table 3. 

The Fisher’s exact test revealed statistically significant (*p* < 0.05) associations between the positivity to at least one VBD and (i) dogs originating from northern Italy (Sites A and B) (*p* = 0.002) and (ii) the exclusive outdoor lifestyles (*p* = 0.022); significant associations were also detected between positivity to at least one TBD and (i) previous tick infestations (*p* = 0.014) and (ii) inadequate prophylactic treatments vs. ticks (*p* < 0.001). Statistically significant associations were also found for single pathogens, i.e., *R. conorii*, *B. canis* and *Anaplasma* spp.; detailed information on the univariate statistical analysis is listed in Table 4. The multivariate logistic regression identified random/irregular applications of ectoparasiticides/anti-feeding products as a risk factor for the exposition to VBDs (*p* = 0.003), with an odds ratio of 3.673 (Table 4). No other risk factors were found.

Positivity to circulating antigens of *D. immitis* and the relative preventative treatment for cardiopulmonary filariosis were not included in the statistical analysis, because only dogs from Sites A and B were from geographic areas where prophylactic treatments are routinely recommended and applied. However, dogs with circulating antigens of *D. immitis*, i.e., 4/242 (1.7%), were not subjected to any kind of preventative treatment. 

## 3. Discussion

Epidemiological, biological and phenological features of the Mediterranean Basin favor the presence and spreading of pathogens and parasites transmitted by vectors and/or intermediate hosts to dogs and cats [15,31,32,33,34,35]. Accordingly, this study confirms that dogs from regions of northern and central Italy are highly exposed to several VBDs.

Regions of southern Italy were not included in this survey, as numerous updated epidemiological data on canine VBD, which show high endemicity, have been published in past years [24,36,37,38,39]. Moreover, flea-borne pathogens were not investigated in this study. This could appear as a shortcoming of the present study, but this survey was focused on TBDs and VBDs by flying insects (mosquitoes and sandflies), which are more frequent and relevant in canine medicine if compared to flea-borne diseases [40].

The results confirm the endemicity of all study VBDs in different regions of Italy. Accordingly, ticks (and fleas) have been found in many dogs and the history of infestations has been declared by several owners. The identification of arthropods collected has been left out of the scope of the present work.

Interesting differences have been found between sites of northern and central Italy in terms of distribution patterns of the study pathogens. The seropositivity values against *R. conorii*, *B. canis* and *Anaplasma* spp. fit with those recorded in other surveys carried out in Italy [14,41,42]. The high level of positivity to *R. conorii* is of high epidemiological relevance, as dogs are useful sentinels for the public health monitoring of spotted fever group rickettsioses and the assessment of the risk of human exposure to *R. conorii*. Nevertheless, serological cross-reactions with other *Rickettsia* species, e.g., *Rickettsia felis* and/or *Rickettsia typhi*, may occur, as discussed elsewhere [42]. Regardless, *R. conorii* remains the most widely distributed *Rickettsia* species in dogs throughout the Mediterranean basin and these results indicate a high exposure to arthropods, which act as vectors of these pathogens [42]. While antibodies against *R. conorii* and *A. phagocytophilum*/*A. platys* have been detected in all sites, no dogs seropositive to *B. canis* were found in sites A and B of northern Italy, with a seroprevalence up to 45% detected in site F of central Italy. This is not surprising, as the main vectors of *R. conorii* and *A. phagocytophilum*, i.e., *Rhipicephalus sanguineus* for *R. conorii* and *A. platys* [43,44], and *Ixodes ricinus* for *A. phagocytophilum* [14], are widespread in northern Italy and present all-year-round [45]. Conversely, *Dermacentor* spp. (*Dermacentor reticulatus*), i.e., the main vector of *B. canis* [46], is scantly distributed in northern Italy with a trend of distribution only in the spring and summer [45]. Thus, lower seroprevalence rates of *B. canis* in sites A and B of northern Italy vs. sites C–F located in central Italy are expected and, accordingly, a decreasing trend of prevalence from central to northern areas has been previously described for this protozoan [47], as shown by the low number of seropositive animals detected in recent studies on kenneled and sheltered dogs from the same areas [42]. It should be noted that kenneled/sheltered dogs, which are at significantly higher risk of infection with *Babesia* spp. [47], were excluded in this survey. Altogether, these factors have likely influenced the absence of dogs seroreacting to *B. canis* in sites A and B of the present study. The positivity values recorded in dogs from sites C to F to *B. canis* can also be explained by a possible cross-reaction between the two large *Babesia* present in Italy, i.e., *B. canis* and *Babesia vogeli*, as the latter is transmitted by *R. sanguineus* and is typically more frequent in central and southern Italy if compared to northern regions [8,48]. Furthermore, cross-reactions can also occur between large and small *Babesia*, as shown for *B. canis* and *Babesia gibsoni* [49] or *Babesia vulpes* [42]. Thus, further considerations on the role of different tick species in transmitting *Babesia* to the study dogs are challenging.

Dogs seropositive for *B. burgdorferi* were found only in sites A and B of northern Italy. This can be explained if one considers that the vector of *B. burgdorferi*, i.e., the castor bean tick *I. ricinus*, is more widespread in northern areas due to the suitable environment and climate, though present also in central and southern Italy [45]. No dogs positive for *B. burgdorferi* were found in a previous study in the same northern regions carried out some years ago [42]. This difference could be explained by the provenance of dogs sampled in that study, i.e., animals in shelters and kennels [42], while mostly hunting dogs were included in northern Italy in the present study. Indeed, hunting dogs are at higher risk to be infested by *I. ricinus,* due to the frequent exposition to wooded areas where this tick species is prevalent [50].

Seropositivity to *Ehrlichia* spp. is the lowest detected in this study. These data are in contrast with the higher infection rates (up to 46.7%) detected in previous surveys carried out in Italy [14,51]. This discrepancy could be explained by cross-reactions between *E. canis* and *A. phagocytophilum* antibodies using the IFAT applied in past studies [14,51], which could have overestimated the dog exposure to *E. canis*. This is supported by data presented in the review by Sainz et al., [14], where a generally higher prevalence of *E. canis* detected using IFAT if compared to the SNAP 4DX has been evidenced. Accordingly, the seroprevalence for *Ehrlichia* spp. detected in this study is in line with values recorded in another seroepidemiological survey carried out in Italy using the SNAP 4Dx some years ago [42].

Dogs with circulating antibodies against *L. infantum* were found in almost all sites, with the exception of Site B. The rates detected in sites E and F fit with those of recent studies carried out in the same regions of central Italy [52,53,54]. The rates found in site C are not comparable with data from a recent study in which similar seroprevalence values were detected but with lower cut-off dilutions [55]. Although only a single dog tested positive for anti-*L. infantum* igG in northern Italy (site A), this finding confirms the stable presence of this protozoan in the north of the country, where the vectors of *L. infantum* are now endemic [56,57]. Colonization by sandflies and dog relocation from South to North Italy contributed to the establishment of the parasite, which should nowadays also be considered endemic in northern areas of Italy [58,59]. Accordingly, endemic foci in Veneto (site A) have been previously described [56]. To the best of the author’s knowledge, this is the first report of *L. infantum* exposure in dogs living in Giglio island (site D). The positive dog had a history of movement to an endemic area of central Italy (i.e., Latium/Continental Tuscany), but it cannot be excluded that the bite of an infected sandfly occurred on the island, as a cat that never moved outside this territory was recently found seropositive for *L. infantum* in the same territory [32]. Therefore, the risk of infection with *L. infantum* should be taken into account for pets travelling to this touristic island and adequate prophylactic treatments are necessary.

The circulating antigen of *D. immitis* was detected in dogs from three of the study sites, i.e., B, C and D. Although this filariid has been traditionally considered endemic only in northern Italy, its prevalence in this area decreased in recent years as a consequence of intensive prophylactic measures [58]. This is confirmed by data obtained in sites A and B. On the other hand, during the past few years, its presence has increased in central and southern Italy due to different factors, including the lack of adequate preventative measures where this parasite is still erroneously considered nonendemic [34,58,60]. The presence of *D. immitis* in sites C and D confirms this trend, which was also suggested by previous surveys [61,62]. Nevertheless, the nematode was not found in sites E and F, where past surveys had shown its occurrence [62,63]. This could likely be due to the low number of dogs examined at a regional level in the present study. As mentioned above for *L. infantum*, this is the first report of *D. immitis* on Giglio island. This result is of interest as it suggests the spread of *D. immitis* in insular and continental [62] areas of the Tuscany region of Central Italy.

Most of the significant statistical associations detected by the Fisher’s exact test were expected. Indeed, a permanently outdoor lifestyle and previous tick infestations are obvious risk factors for TBDs. Although dogs living in northern Italy were apparently more at risk of TBDs, the statistical association is likely biased by the inclusion of several hunting dogs from sites A and B, which are particularly prone to be infested by ectoparasites and infected by VBDs [36]. The irregular/random use of ectoparasiticides and anti-feeding products is a crucial risk factor. In fact, out of 114 dogs irregularly treated, 68 (59.6%) were positive for at least one VBD and 84 (73.6%) had a history of tick infestation. This confirms that the constant, regular and timely use of ectoparasiticides/anti-feeding products is pivotal for preventing VBDs in dogs. Unexpectedly, past and current flea infestations were significantly associated with seropositivity to *B. canis* and *Anaplasma* spp. respectively. As these pathogens are not transmitted by fleas, this could be explained by a general lack of adherence of dog owners to the veterinary recommendation to use appropriate medications against arthropods. In fact, during the enrollment of dogs of the present studies, many owners declared to treat their animal only “if necessary”, i.e., when ticks or fleas were already present on their dogs and could have already transmitted pathogens. Accordingly, 28/30 dogs with fleas at the time of sampling received inadequate prophylactic treatments. Fifteen of them were seropositive for at least 1 TBD, and for 13 of them, owners referred a history of previous tick infestation, indicating that dogs presenting with fleas were also not protected against ticks. This is not surprising, as many formulations are efficacious against both arthropods and an inadequate protection against ticks may also favor the presence of fleas and possible transmitted diseases. In addition, the three dogs with ticks at the time of bleeding did not receive regular and adequate treatments and all of them tested positive for TBDs. 

The present data confirm that dogs are highly exposed to VBDs in Italy despite the extensive use of ectoparasiticides and anti-feeding products, i.e., all study dog owners declared the use of medications vs. vectors. The owner compliance proved to be crucial to protect dogs from VBDs in endemic areas. These results show little general adherence to adequate treatment regimens as the majority of dogs did not receive adequate treatments in terms of parasiticide timing and schedules to protect the animals from ticks, fleas and sandflies/mosquitoes infestations. Thus, it is evidenced that adequate preventative measures are realistically overlooked not only in stray and kenneled dogs [64,65], but also in owned dogs, thus increasing the risk of ectoparasite infestations and transmitted diseases and favoring the presence of infected arthropods in domestic environments and households.

While the incorrect/irregular use of medications was clearly correlated to the exposure of VBDs, the rates of positivity were also found in dogs whose owners declared accurate control programs. These studies are based only on statements made by the owners, which cannot be confirmed with sound evidence. In this regard, it should be taken into account that the correct use of parasiticide by owners can be impaired by the cost of the product and inadequate knowledge on the correct mode and frequency of product application, e.g., impact of wetting/bathing the dog [66,67]. The characteristics of different formulations used by the owners could also have an impact, as they may have varying durations of efficacy against ticks or fleas or sandflies/mosquitoes, e.g., deltamethrin or imidacloprid/flumethrin collars, spot-on imidacloprid/permethrin or fipronil/permethrin, or against different species of the same arthropod class, e.g., oral fluralaner, spot-on imidacloprid/permethrin, or fipronil/permethrin. One relevant example is given by the fact that some ectoparasite anti-feeding products have an efficacy lasting 2 or 3 weeks against sandflies (depending on the phlebotomine species), while others present a mean 4-week efficacy against fleas and ticks. Thus, misinformed and inattentive owners who apply such products with a 4-week interval leave their dogs unprotected against sandflies for 1 or 2 weeks, enhancing the risk of *L. infantum* infection. Additionally, different products have a different killing speed against arthropod vectors that start to feed on treated animals. Such a difference impacts the ability to prevent diseases caused by pathogens that are transmitted at different timepoints during the blood meal. Altogether, this variability may explain the relatively high proportion of study dogs that scored seropositive to one or more pathogens despite an “all over the year” administration of prophylactic treatment.

Other than a general lack of adherence to veterinary recommendations and guidelines, it should be taken into account that the relatively high overall seroexposure rate to CVBDs reported in this study could be due to a need to amend the frequency of use of products (e.g., seasonal vs. all-year-round, shorter intervals). For instance, global warming has the potential to alter the spatial-temporal distribution of CVBDs and to influence the life cycle, reproduction rates, and survival of vectors, thus triggering the occurrence and abundance of the pathogens they transmit in given areas in larger territories and for longer seasons [20,21]. 

Seropositive dogs may also harbor one or more pathogens even when clinically healthy, thus acting as a source of infection for the vectors. Therefore, ectoparasiticides/anti-feeding products should also be administered to positive dogs, to reduce the likelihood to infect vectors and minimize the risk of transmission to other hosts. This is of importance also considering that the same arthropods can transmit different pathogens, some of them with a zoonotic potential. Therefore, the protection of positive animals is crucial to avoid mixed infections, which are highly common in areas endemic for canine VBDs [15,42], as also shown by the high number of dogs simultaneously exposed to more VBDs.

These aspects play a relevant role in the epidemiology of VBDs, with both veterinary and Public Health implications. While many of the VBDs investigated in this survey are of utmost importance mostly in canine medicine, e.g., *L. infantum*, *B. canis*, *E. canis* and *D. immitis*, dogs can act as sources of vector infection for *L. infantum* (causing human visceral leishmaniosis) and for VBDs of secondary veterinary impact that can instead cause severe, and in some cases, life-threatening disease in humans. This is the case of *R. conorii*, *B. burgdorferi*, and *A. phagocytophilum* causing Mediterranean Spotted Fever, Lyme Borreliosis and Granulocytic Anaplasmosis, respectively [68,69,70,71]. 

In conclusion, this study provides new knowledge on the occurrence of VBDs in dogs that are usually treated against ectoparasites and subjected to diverse treatment schedules. Similar data are few and new large-scale studies aiming to evaluate the efficacy on different prophylactic treatments are encouraged, as they represent a reliable basis to improve and implement current control programs. Indeed, the present results highlight that control regimens may be erroneously put in place in several regions of Italy despite compliance claimed by owners, and that a higher level of awareness by both owners and veterinarians is needed. A continuous monitoring and surveillance of health hazards posed by dogs exposed to infected vectors is crucial toward the correct use of preventative medications against ectoparasites. Efficacious regimens are of paramount importance to control the occurrence and distribution of VBDs and to protect animals and humans. A plethora of products are commercially available, and veterinarians must educate pet owners to conduct routine check-ups for VBDs and to appropriate control programs selected on a case-by-case basis and according to local epidemiological scenarios.

Moreover, several broad-spectrum parasiticide formulations may permit at the same time the control of arthropod vectors and of internal parasites of dogs, including those with a zoonotic potential. Given that canine endoparasites are widespread in dog populations of Mediterranean countries, where CVBDs are endemic, and may extensively contaminate the environment [15,42,72,73,74,75], improving the awareness and continuing the education of veterinarian and pet owners on the use of broad-spectrum parasiticides are priorities in veterinary medicine.

## 4. Materials and Methods

### 4.1. Study Design, Areas, and Sampling 

**Figure 1:**
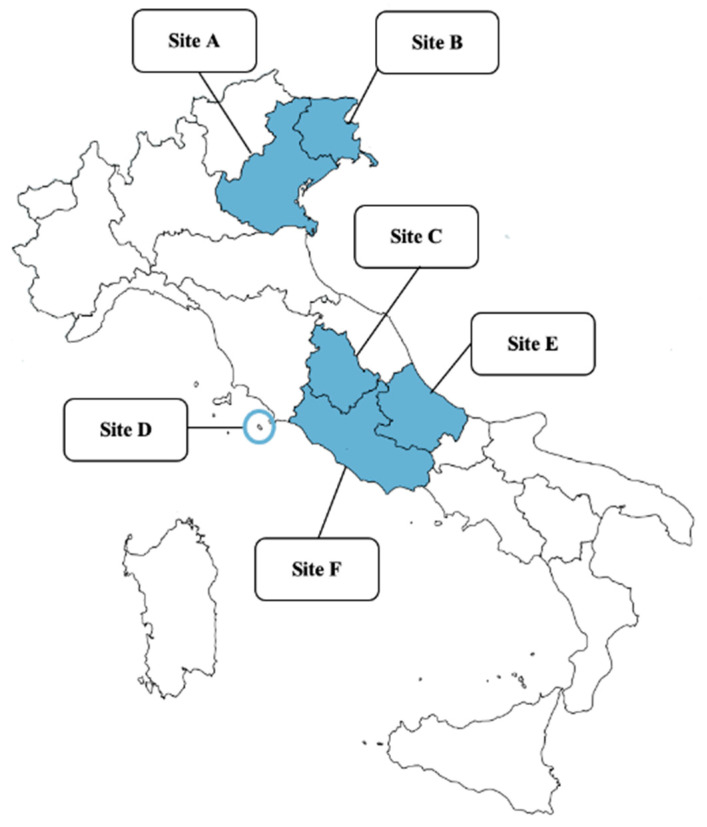
Map of Italy: the study areas of the present study are indicated. Northern Italy, Veneto (Site A); Friuli-Venezia Giulia (Site B); Central Italy, Umbria (Site C); Giglio Island, Tuscany (Site D); Abruzzo (Site E); Latium (Site F).

A total of 242 privately owned and apparently healthy dogs from different regions of northern and central Italy (Figure 1) were included in the study, i.e., 33, 34, 45, 42, 48, and 40 from sites A (Veneto), B (Friuli-Venezia Giulia), C (Umbria), D (Giglio Island, Tuscany), E (Abruzzo), and F (Latium), respectively. 

The study was performed in the framework of routine medical checks coordinated by local veterinarians and dogs were selected as a convenience dataset based on the following criteria: (i) willingness of the owners to participate; (ii) habitat in endemic areas; (iii) at least one vector season experienced; (iv) under control for ectoparasites. Signalment and anamnesis, including data on age, sex, breed, lifestyle, travel history, cohabitation, and/or contact with other animals, class, timing and schedule of antiparasitic treatment and previous history of ectoparasite infestations were registered for each study animal. 

A consent form was signed by the owner before sample collection. Blood samples were obtained individually through the venipuncture of the jugular, cephalic or saphenous veins, and they were transferred in a tube without anticoagulant, kept at room temperature until clot formation and centrifuged to separate the serum. Each veterinarian in charge of the study in each site examined the full body surface and hair of the study dogs with conventional parasitological methods to detect the presence of ectoparasites. 

Dogs’ data were broken down according to groups based on timing and medications administered. More in detail, treatments were considered adequate or inadequate in the prevention of TBDs and leishmaniosis, considering the biology and the ecology of vectors. Moreover, dogs were divided into four categories based on the timing of the treatment schedules, i.e., dogs (i) receiving treatments all-year-round, (ii) from spring to autumn, (iii) only in summer and (iv) random/irregular treatments.

### 4.2. Serology

Each serum sample was subjected to the following different serological examinations, according to the manufacturer instructions: 

- SNAP 4DX (IDEXX Laboratories, Inc., Westbrook, ME, USA) for the detection of *D. immitis* circulating antigen and of antibodies against *A. phagocytophilum*/*A. platys*, *E. canis/E. ewingii*, and *B. burgdorferi*

- Indirect immunofluorescence antibody test (IFAT), using a commercially available kit to detect antibodies (IgG) against *L. infantum* (MegaFLUO Leish-Megacor Diagnostik GmbH), *B. canis* (MegaFLUO BABESIA *canis*-Megacor Diagnostik GmbH), and *R. conorii* (MegaFLUO RICKETTSIA *conorii*-Megacor Diagnostik GmbH). The screening dilutions applied were 1:100, 1:160, and 1:64, respectively, according to the manufacturer instructions.

Possible cross-reactions between (i) *R. conorii* and *R. felis* and/or *R. typhi*, [42] (ii) *B. canis* and other large (*B. vogeli*) or small (*B. gibsoni* and/or *B. vulpes*) [42,48,49], and (iii) *E. canis* and *A. phagocytophilum* [14,41] have been taken into proper account and discussed. 

### 4.3. Data Analysis

A statistical analysis was performed using GraphPad Prism 9 (GraphPad Software, LLC). The Fisher’s exact test was used to evaluate the presence of significant associations (*p* < 0.05) between exposure to VBDs and possible risk factors. A multivariate logistic regression was also performed to identify possible risk factors. The strength of eventual risk factors identified was measured using the odds ratio (OR), and the 95% confidence interval was calculated.

## Figures and Tables

**Table 1:** Results of serological examinations (SNAP 4DX rapid test and Immunofluorescence Antibody Test, IFAT): number/total (n/tot) and percentage (%) of dogs positive for different pathogens in Italy ^§^.

	SNAP 4DX				IFAT				
Site	*An * n/tot (%)	*Eh * n/tot (%)	*Bb* n/tot (%)	*Di* n/tot (%)	*Li* n/tot (%)	*Rc* n/tot (%)	*Bc* n/tot (%)	Mixed n/tot ** (%)	Total n/tot * (%)
**A**	7/33 (21.2)	- -	3/33 (9.1)	- -	1/33 (3.0)	19/33 (57.6)	- -	7/33 (21.2)	22/33 (66.7)
**B**	1/34 (2.9)	- -	1/34 (2.9)	2/34 (5.9)	- -	22/34 (64.7)	- -	3/34 (8.8)	23/34 (67.6)
**C**	1/45 (2.2)	- -	- -	1/45 (2.2)	3/45 (6.7)	5/45 (11.1)	2/45 (4.4)	2/45 (4.4)	10/45 (22.2)
**D**	3/42 (7.1)	- -	- -	1/42 (2.4)	1/42 (2.4)	13/42 (31.0)	- -	1/42 (2.4)	17/42 (40.5)
**E**	4/48 (8.3)	1/48 (2.1)	- -	- -	4/48 (8.3)	13/48 (27.1)	5/48 (10.4)	4/48 (8.3)	21/48 (43.8)
**F**	6/40 (15.0)	- -	- -	- -	2/40 (5.0)	26/40 (65.0)	18/40 (45.0)	17/40 (42.5)	31/40 (77.5)
**Total** **n/tot (%)**	22/242 (9.1)	1/242 (0.4)	4/242 (1.7)	4/242 (1.7)	11/242 (4.5)	98/242 (40.5)	25/242 (10.3)	34/242 (14.0)	124/242 (51.2)

* Dogs positive to at least one pathogen. ***An***: *Anaplasma* spp.; ***Eh***: *Ehrlichia* spp.; ***Bb***: *Borrelia burgdorferi*; ***Di***: *Dirofilaria immitis*; ***Li***: *Leishmania infantum*; ***Rc***: *Rickettsia conorii*; ***Bc***: *Babesia canis*. ** Dogs that tested positive to two or more pathogens investigated in this study. ^§^ Sites of northern (i.e., Site A: Veneto and Site B: Friuli-Venezia Giulia) and central Italy (i.e., Site C: Umbria, Site D: Giglio Island, Tuscany, Site E: Abruzzo, and Site F: Latium).

**Table 2:** Number/total (n/tot) and percentage (%) of dogs with current or previous flea and tick infestations and related seropositivity detected upon SNAP 4DX rapid test and immunofluorescence antibody test (IFAT).

		SNAP 4DX				IFAT				
Ectoparasite infestations	n/tot (%)	*An*	*Eh*	*Bb*	*Di*	*Li*	*Rc*	*Bc*	Mixed	Total
		n/tot (%)	n/tot (%)	n/tot (%)	n/tot (%)	n/tot (%)	n/tot (%)	n/tot (%)	n/tot ** (%)	n/tot * (%)
**Flea (current)**	30 (12.4%)	2/30	*-*	*-*	*-*	2/30	13/30	8/30	8/30	16/30
		(6.7)	*-*	*-*	*-*	(6.7)	(43.3)	(26.7)	(26.7)	(53.3)
**Flea (past)**	73 (30.2%)	11/73	-	-	1/73	5/73	33/73	16/73	18/73	40/73
		(15.0)	-	-	(1.3)	(6.8)	(45.2)	(21.9)	(24.6)	(54.8)
**Ticks (current)**	3 (1.2%)	-	-	-	-	-	3/3	1/3	1/3	3/3
		-	-	-	-	-	(100)	(33.3)	(33.30	(100)
**Ticks (past)**	128 (52.9%)	18/128	-	3/128	4/128	4/128	68/128	16/128	29/128	79/128
		(14.1)	-	(2.3)	(3.1)	(3.1)	(53.1)	(12.5)	(22.7)	(61.7)

* Dogs positive to at least one pathogen. ***An***: *Anaplasma* spp.; ***Eh***: *Ehrlichia* spp.; ***Bb***: *Borrelia burgdorferi*; ***Di***: *Dirofilaria immitis*; ***Li***: *Leishmania infantum*; ***Rc***: *Rickettsia conorii*; ***Bc***: *Babesia canis*. ** dogs that tested positive to two or more pathogens investigated in this study.

**Table 3:** Number/total (n/tot) and percentage (%) of dogs subjected to different preventative treatment schedules with different formulations (mono-products or broad-spectrum medications) and seropositivity detected at the SNAP 4DX rapid test and immunofluorescence antibody test (IFAT).

			SNAP 4DX				IFAT			
Formulations n (%)	Treatment Schedule	n/tot (%)	*An * n/tot (%)	*Eh* n/tot (%)	*Bb* n/tot (%)	*Di* n/tot (%)	*Li* n/tot (%)	*Rc* n/tot (%)	*Bc* n/tot (%)	Total n/tot * (%)
Pyrethroids 120 (49.6)	Throughout the year	46/120 (38.3)	1/46 (2.8)	-	-	-	3/46 (6.5)	10/46 (21.7)	4/46 (8.7)	16/46 (34.8)
	From spring to autumn	44/120 (36.6)	1/44 (2.3)	-	-	1/44 (2.3)	1/44 (2.3)	13/44 (29.5)	3/44 (6.8)	19/44 (43.2)
	Only in summer	13/120 (10.8)	1/13 (7.7)	-	1/13 (7.7)	-	-	4/13 (30.8)	-	6/13 (46.2)
	Irregular	17/120 (14.2)	2/17 (11.8)	-	2/17 (11.8)	1/17 (5.9)	1/17 (5.9)	7/17 (41.2)	-	11/17 (64.7)
Isoxazolines 36 (14.9)	Throughout the year	15/36 (41.7)	4/15 (26.7)	1/15 (6.6)	-	-	2/15 (13.3)	8/15 (53.3)	-	10/15 (66.7)
	From spring to autumn	2/36 (5.6)	-	-	-	-	-	1/2 (50.0)	-	1/2 (50.0)
	Only in summer	1/36 (2.8)	-	-	-	-	-	-	-	-
	Irregular	18/36 (50)	6/18 (33.3)	-	-	1/18 (5.5)	-	11/18 (61.1)	-	11/18 (61.1)
Fenilpirazoles 27 (11.1)	From spring to autumn	2/27 (7.4)	-	-	-	-	-	1/2 (50.0)	-	1/2 (50.0)
	Only in summer	5/27 (18.5)	1/5 (20.0)	-	-	-	-	3/5 (60.0)	-	3/5 (60.0)
	Irregular	20/27 (74.0)	-	-	-	-	1/20 (5.0)	8/20 (40.0)	2/20 (10.0)	11/20 (55.0)
Organophosphorus insecticides 42 (17.3)	Irregular	42/42 (100)	6/42 (14.3)	-	-	-	2/42 (4.8)	28/42 (66.7)	15/42 (35.7)	30/42 (71.4)
Unknown product 17 (7)	Irregular	17/17 (100)	-	-	-	1/17 (5.9)	-	4/17 (23.5)	-	5/17 (29.4)

* Dogs positive to at least one pathogen. ***An***: *Anaplasma* spp.; ***Eh***: *Ehrlichia* spp.; ***Bb***: *Borrelia burgdorferi*; ***Di***: *Dirofilaria immitis*; ***Li***: *Leishmania infantum*; ***Rc***: *Rickettsia conorii*; ***Bc***: *Babesia canis*.

**Table 4:** Statistically significant associations found between the seropositivity to at least one vector-borne pathogen (VBP), at least one tick-borne pathogen (TBP), or individual pathogens, and possible risk factors (Fisher’s exact test). The results of the risk factor analysis (multivariate logistic regression) are also shown.

Fisher’s Exact Test			
	*p* Value	OR *	95% CI *
**At least one VBP**			
Living in Northern Italy	0.002	2.486	1.356–4.445
Outdoor lifestyle	0.022	1.870	1.079–3.194
**At least one TBP**			
Inadequate tick prevention	<0.001	1.939	1.171–3.267
Previous tick infestation	0.014	2.793	1.661–4.662
***Rickettsia conorii***			
Living in Northern Italy	<0.001	3. 265	1.783–5.746
Outdoor lifestyle	<0.001	2.720	1.531–4.767
Previous tick infestation	<0.001	3.173	1.826–5.334
***Anaplasma* spp.**			
Previous tick infestation	0.006	4.500	1.472–12.55
Previous flea infestation	0.049	2. 548	1.081–5.979
***Babesia canis***			
Current flea infestation	0.005	4.171	1.612–10.04
Previous flea infestation	<0.001	4.990	2.144–11.85
**Multivariate Logistic Regression**			
**At least one VBP**			
Random/irregular treatment	0.003	3.673	1.077–12.53

* OR = odds ratio; **CI** = confidence interval.

## Data Availability

All Study data are presented in the article.

## References

[1] Beugnet F., Marié J.L. (2009). Emerging arthropod-borne diseases of companion animals in Europe. Vet. Parasitol..

[2] Otranto D., Dantas-Torres F., Breitschwerdt E.B. (2009). Managing canine vector-borne diseases of zoonotic concern: Part one. Trends Parasitol..

[3] Self S.C.W., Liu Y., Nordone S.K., Yabsley M.J., Walden H.S., Lund R.B., Bowman D.D., Carpenter C., McMahan C.S., Gettings J.R. (2019). Canine vector-borne disease: Mapping and the accuracy of forecasting using big data from the veterinary community. Anim. Health Res. Rev..

[4] Jefferies R., Ryan U.M., Muhlnickel C.J., Irwin P.J. (2003). Two species of canine *Babesia* in Australia: Detection and characterization by PCR. J. Parasitol..

[5] Simón F., Siles-Lucas M., Morchón R., González-Miguel J., Mellado I., Carretón E., Montoya-Alonso J.A. (2012). Human and animal dirofilariasis: The emergence of a zoonotic mosaic. Clin. Microbiol. Rev..

[6] Gharbi M., Mhadhbi M., Rejeb A., Jaouadi K., Rouatbi M., Darghouth M.A. (2015). Leishmaniosis (*Leishmania infantum* infection) in dogs. Rev. Sci. Technol. Off. Int. Epiz..

[7] Singh M.N., Raina O.K., Sankar M., Rialch A., Tigga M.N., Kumar G.R., Banerjee P.S. (2016). Molecular detection and genetic diversity of *Babesia gibsoni* in dogs in India. Infect. Genet. Evol..

[8] Solano-Gallego L., Sainz Á., Roura X., Estrada-Peña A., Miró G. (2016). A review of canine babesiosis: The European perspective. Parasit. Vectors.

[9] Barash N.R., Thomas B., Birkenheuer A.J., Breitschwerdt E.B., Lemler E., Qurollo B.A. (2019). Prevalence of *Babesia* spp. and clinical characteristics of *Babesia vulpes* infections in North American dogs. J. Vet. Intern. Med..

[10] Guo W.P., Xie G.C., Li D., Su M., Jian R., Du L.Y. (2020). Molecular detection and genetic characteristics of *Babesia gibsoni* in dogs in Shaanxi Province, China. Parasit. Vectors.

[11] McCall J.W., Genchi C., Kramer L.H., Guerrero J., Venco L. (2008). Heartworm disease in animals and humans. Adv. Parasitol..

[12] Paltrinieri S., Solano-Gallego L., Fondati A., Lubas G., Gradoni L., Castagnaro M., Crotti A., Maroli M., Oliva G., Roura X. (2010). Guidelines for diagnosis and clinical classification of leishmaniasis in dogs. J. Am. Vet. Med. Assoc..

[13] Strobl A., Künzel F., Tichy A., Leschnik M. (2020). Complications and risk factors regarding the outcomes of canine babesiosis in Central Europe—A retrospective analysis of 240 cases. Acta Vet. Hung..

[14] Sainz Á., Roura X., Miró G., Estrada-Peña A., Kohn B., Harrus S., Solano-Gallego L. (2015). Guideline for veterinary practitioners on canine ehrlichiosis and anaplasmosis in Europe. Parasit. Vectors.

[15] Diakou A., Di Cesare A., Morelli S., Colombo M., Halos L., Simonato G., Tamvakis A., Beugnet F., Paoletti B., Traversa D. (2019). Endoparasites and vector-borne pathogens in dogs from Greek islands: Pathogen distribution and zoonotic implications. PLoS Negl. Trop. Dis..

[16] Springer A., Glass A., Topp A.K., Strube C. (2020). Zoonotic Tick-Borne Pathogens in Temperate and Cold Regions of Europe—A Review on the Prevalence in Domestic Animals. Front. Vet. Sci..

[17] Little S., Braff J., Place J., Buch J., Dewage B.G., Knupp A., Beall M. (2021). Canine infection with *Dirofilaria immitis*, *Borrelia burgdorferi*, *Anaplasma* spp., and *Ehrlichia* spp. in the United States, 2013-2019. Parasit. Vectors.

[18] Otranto D., Dantas-Torres F., Mihalca A., Traub R., Lappin M., Baneth G. (2017). Zoonotic parasites of sheltered and stray dogs in the era of the global economic and political crisis. Trends Parasitol..

[19] Wright I., Jongejan F., Marcondes M., Peregrine A., Baneth G., Bourdeau P., Bowman D.D., Breitschwerdt E.B., Capelli G., Cardoso L. (2020). Parasites and vector-borne diseases disseminated by rehomed dogs. Parasit. Vectors.

[20] Beugnet F., Chalvet-Monfray K. (2013). Impact of climate change in the epidemiology of vector-borne diseases in domestic carnivores. Comp. Immunol. Microbiol. Infect. Dis..

[21] Ogden N.H., Lindsay L.R. (2016). Effects of Climate and Climate Change on Vectors and Vector-Borne Diseases: Ticks Are Different. Trends Parasitol..

[22] Díaz-Regañón D., Roura X., Suárez M.L., León M., Sainz Á. (2020). Serological evaluation of selected vector-borne pathogens in owned dogs from northern Spain based on a multicenter study using a commercial test. Parasit. Vectors.

[23] Kostopoulou D., Gizzarelli M., Ligda P., Foglia Manzillo V., Saratsi K., Montagnaro S., Schunack B., Boegel A., Pollmeier M., Oliva G. (2020). Mapping the canine vector-borne disease risk in a Mediterranean area. Parasit. Vectors.

[24] Petruccelli A., Ferrara G., Iovane G., Schettini R., Ciarcia R., Caputo V., Pompameo M., Pagnini U., Montagnaro S. (2020). Seroprevalence of *Ehrlichia* spp., *Anaplasma* spp., *Borrelia burgdorferi* sensu lato, and *Dirofilaria immitis* in Stray Dogs, from 2016 to 2019, in Southern Italy. Animals.

[25] Diakou A., Di Cesare A., Accettura P.M., Barros L., Iorio R., Paoletti B., Frangipane di Regalbono A., Halos L., Beugnet F., Traversa D. (2017). Intestinal parasites and vector-borne pathogens in stray and free-roaming cats living in continental and insular Greece. PLoS Negl. Trop. Dis..

[26] Morelli S., Crisi P.E., Di Cesare A., De Santis F., Barlaam A., Santoprete G., Parrinello C., Palermo S., Mancini P., Traversa D. (2019). Exposure of client-owned cats to zoonotic vector-borne pathogens: Clinic-pathological alterations and infection risk analysis. Comp. Immunol. Microbiol. Infect. Dis..

[27] Neves M., Lopes A.P., Martins C., Fino R., Paixão C., Damil L., Lima C., Alho A.M., Schallig H.D.F.H., Dubey J.P. (2020). Survey of *Dirofilaria immitis* antigen and antibodies to *Leishmania infantum* and *Toxoplasma gondii* in cats from Madeira Island, Portugal. Parasit. Vectors.

[28] Dantas-Torres F., Otranto D. (2016). Best Practices for Preventing Vector-Borne Diseases in Dogs and Humans. Trends Parasitol..

[29] Lavan R.P., Tunceli K., Zhang D., Normile D., Armstrong R. (2017). Assessment of dog owner adherence to veterinarians’ flea and tick prevention recommendations in the United States using a cross-sectional survey. Parasit. Vectors.

[30] Lavan R., Armstrong R., Tunceli K., Normile D. (2018). Dog owner flea/tick medication purchases in the USA. Parasit. Vectors.

[31] Morelli S., Colombo M., Dimzas D., Barlaam A., Traversa D., Di Cesare A., Russi I., Spoletini R., Paoletti B., Diakou A. (2020). *Leishmania infantum* Seroprevalence in Cats From Touristic Areas of Italy and Greece. Front. Vet. Sci..

[32] Morelli S., Diakou A., Di Cesare A., Schnyder M., Colombo M., Strube C., Dimzas D., Latino R., Traversa D. (2020). Feline lungworms in Greece: Copromicroscopic, molecular and serological study. Parasitol. Res..

[33] Di Cesare A., Morelli S., Colombo M., Simonato G., Veronesi F., Marcer F., Diakou A., D’Angelosante R., Pantchev N., Psaralexi E. (2020). Is Angiostrongylosis a Realistic Threat for Domestic Cats?. Front. Vet. Sci..

[34] Panarese R., Iatta R., Latrofa M.S., Zatelli A., Ćupina A.I., Montarsi F., Pombi M., Mendoza-Roldan J.A., Beugnet F., Otranto D. (2020). Hyperendemic *Dirofilaria immitis* infection in a sheltered dog population: An expanding threat in the Mediterranean region. Int. J. Parasitol..

[35] Montoya-Alonso J.A., Morchón R., Costa-Rodríguez N., Matos J.I., Falcón-Cordón Y., Carretón E. (2020). Current Distribution of Selected Vector-Borne Diseases in Dogs in Spain. Front. Vet. Sci..

[36] Piantedosi D., Neola B., D’Alessio N., Di Prisco F., Santoro M., Pacifico L., Sgroi G., Auletta L., Buch J., Chandrashekar R. (2017). Seroprevalence and risk factors associated with *Ehrlichia canis*, *Anaplasma* spp., *Borrelia burgdorferi* sensu lato, and *D. immitis* in hunting dogs from southern Italy. Parasitol. Res..

[37] Veneziano V., Piantedosi D., Ferrari N., Neola B., Santoro M., Pacifico L., Sgroi G., D’Alessio N., Panico T., Leutenegger C.M. (2018). Distribution and risk factors associated with *Babesia* spp. infection in hunting dogs from Southern Italy. Ticks Tick Borne Dis..

[38] Gizzarelli M., Foglia Manzillo V., Ciuca L., Morgoglione M.E., El Houda Ben Fayala N., Cringoli G., Oliva G., Rinaldi L., Maurelli M.P. (2019). Simultaneous Detection of Parasitic Vector Borne Diseases: A Robust Cross-Sectional Survey in Hunting, Stray and Sheep Dogs in a Mediterranean Area. Front. Vet. Sci..

[39] Migliore S., Gargano V., De Maria C., Gambino D., Gentile A., Vitale Badaco V., Schirò G., Mira F., Galluzzo P., Vicari D. (2020). A Cross Sectional Study on Serological Prevalence of *Ehrlichia canis* and *Rickettsia conorii* in Different Canine Population of Sicily (South-Italy) during 2017–2019. Animals.

[40] Day M. (2016). Cats are not small dogs: Is there an immunological explanation for why cats are less affected by arthropod-borne disease than dogs?. Parasit. Vectors.

[41] Olivieri E., Zanzani S.A., Latrofa M.S., Lia R.P., Dantas-Torres F., Otranto D., Manfredi M.T. (2016). The southernmost foci of *Dermacentor reticulatus* in Italy and associated *Babesia canis* infection in dogs. Parasit. Vectors.

[42] Traversa D., Di Cesare A., Simonato G., Cassini R., Merola C., Diakou A., Halos L., Beugnet F., Frangipane di Regalbono A. (2017). Zoonotic intestinal parasites and vector-borne pathogens in Italian shelter and kennel dogs. Comp. Immunol. Microbiol. Infect. Dis..

[43] Socolovschi C., Gaudart J., Bitam I., Huynh T.P., Raoult D., Parola P. (2012). Why are there so few *Rickettsia conorii*-infected *Rhipicephalus sanguineus* ticks in the wild?. PLoS Negl. Trop. Dis..

[44] Snellgrove A.N., Krapiunaya I., Ford S.L., Stanley H.M., Wickson A.G., Hartzer K.L., Levin M.L. (2020). Vector competence of *Rhipicephalus sanguineus* sensu stricto for *Anaplasma platys*. Ticks Tick Borne Dis..

[45] Maurelli M.P., Pepe P., Colombo L., Armstrong R., Battisti E., Morgoglione M.E., Counturis D., Rinaldi L., Cringoli G., Ferroglio E. (2018). A national survey of Ixodidae ticks on privately owned dogs in Italy. Parasit. Vectors.

[46] Rubel F., Brugger K., Pfeffer M., Chitimia-Dobler L., Didyk Y.M., Leverenz S., Dautel H., Kahl O. (2016). Geographical distribution of *Dermacentor marginatus* and *Dermacentor reticulatus* in Europe. Ticks Tick Borne Dis..

[47] Otranto D., Dantas-Torres F. (2010). Canine and feline vector-borne diseases in Italy: Current situation and perspectives. Parasit. Vectors.

[48] Solano-Gallego L., Trotta M., Carli E., Carcy B., Caldin M., Furlanello T. (2008). *Babesia canis canis* and *Babesia canis vogeli* clinicopathological findings and DNA detection by means of PCR-RFLP in blood from Italian dogs suspected of tick-borne disease. Vet. Parasitol..

[49] Yamane I., Thomford J.W., Gardner I.A., Dubey J.P., Levy M., Conrad P.A. (1993). Evaluation of the indirect fluorescent antibody test for diagnosis of *Babesia gibsoni* infections in dogs. Am. J. Vet. Res..

[50] Ehrmann S., Liira J., Gärtner S., Hansen K., Brunet J., Cousins S.A.O., Deconchat M., Decocq G., De Frenne P., De Smedt P. (2017). Environmental drivers of *Ixodes ricinus* abundance in forest fragments of rural European landscapes. BMC Ecol..

[51] Ebani V.V. (2019). Serological Survey of *Ehrlichia canis* and *Anaplasma phagocytophilum* in Dogs from Central Italy: An Update (2013–2017). Pathogens.

[52] Sauda F., Malandrucco L., Macrì G., Scarpulla M., De Liberato C., Terracciano G., Fichi G., Berrilli F., Perrucci S. (2018). *Leishmania infantum*, *Dirofilaria* spp. and other endoparasite infections in kennel dogs in central Italy. Parasite.

[53] Rombolà P., Barlozzari G., Carvelli A., Scarpulla M., Iacoponi F., Macrì G. (2021). Seroprevalence and risk factors associated with exposure to *Leishmania infantum* in dogs, in an endemic Mediterranean region. PLoS ONE.

[54] De Massis F., Ippoliti C., Simona I., Tittarelli M., Pelini S., Giansante D., Ciarrocchi A. (2020). Canine Leishmaniasis: Serological Results in Private and Kennel Dogs Tested over a Six-Year Period (2009–2014) in Abruzzo and Molise Regions, Italy. Microorganisms.

[55] Kostalova T., Lestinova T., Maia C., Sumova P., Vlkova M., Willen L., Polanska N., Fiorentino E., Scalone A., Oliva G. (2017). The recombinant protein rSP03B is a valid antigen for screening dog exposure to *Phlebotomus perniciosus* across foci of canine leishmaniasis. Med. Vet. Entomol..

[56] Morosetti G., Bongiorno G., Beran B., Scalone A., Moser J., Gramiccia M., Gradoni L., Maroli M. (2009). Risk assessment for canine leishmaniasis spreading in the north of Italy. Geospat. Health.

[57] Signorini M., Cassini R., Drigo M., Frangipane di Regalbono A., Pietrobelli M., Montarsi F., Stensgaard A.S. (2014). Ecological niche model of *Phlebotomus perniciosus,* the main vector of canine leishmaniasis in north-eastern Italy. Geospat. Health.

[58] Mendoza-Roldan J., Benelli G., Panarese R., Iatta R., Furlanello T., Beugnet F., Zatelli A., Otranto D. (2020). *Leishmania infantum* and *Dirofilaria immitis* infections in Italy, 2009-2019: Changing distribution patterns. Parasit. Vectors.

[59] Michelutti A., Toniolo F., Bertola M., Grillini M., Simonato G., Ravagnan S., Montarsi F. (2021). Occurrence of Phlebotomine sand flies (Diptera: Psychodidae) in the northeastern plain of Italy. Parasit. Vectors.

[60] Santoro M., Miletti G., Vangone L., Spadari L., Reccia S., Fusco G. (2019). Heartworm disease *(Dirofilaria immitis*) in two roaming dogs from the urban area of Castel Volturno, Southern Italy. Front. Vet. Sci..

[61] Piergili-Fioretti D., Diaferia M., Grelloni V., Maresca C. (2003). Canine filariosis in Umbria: An update of the occurrence one year after the first observation of autochthonous foci. Parassitologia.

[62] Macchioni F., Sed G., Cecchi F. (2020). Canine filarial infections in an area of Central Italy (Tuscany-Latium border) historically free from the disease. Vet. Parasitol. Reg. Stud. Rep..

[63] Traversa D., Aste G., Milillo P., Capelli G., Pampurini F., Tunesi C., Santori D., Paoletti B., Boari A. (2010). Autochthonous foci of canine and feline infections by *Dirofilaria immitis* and *Dirofilaria repens* in central Italy. Vet. Parasitol..

[64] Baneth G., Thamsborg S.M., Otranto D., Guillot J., Blaga R., Deplazes P., Solano-Gallego L. (2016). Major Parasitic Zoonoses Associated with Dogs and Cats in Europe. J. Comp. Pathol..

[65] Otranto D. (2018). Arthropod-borne pathogens of dogs and cats: From pathways and times of transmission to disease control. Vet. Parasitol..

[66] Elsheikha E. (2016). Flea and tick control: Innovative approaches to owner compliance. Vet. Times.

[67] Elsheikha H. (2017). Ectoparasites: Preventive plans and innovations in treatment. Vet. Times.

[68] Jin H., Wei F., Liu Q., Qian J. (2012). Epidemiology and control of human granulocytic anaplasmosis: A systematic review. Vector Borne Zoonotic Dis..

[69] Portillo A., Santibáñez S., García-Álvarez L., Palomar A.M., Oteo J.A. (2015). Rickettsioses in Europe. Microbes Infect..

[70] Littman M.P., Gerber B., Goldstein R.E., Labato M.A., Lappin M.R., Moore G.E. (2018). ACVIM consensus update on Lyme borreliosis in dogs and cats. J. Vet. Intern. Med..

[71] Miró G., López-Vélez R. (2018). Clinical management of canine leishmaniosis versus human leishmaniasis due to *Leishmania infantum*: Putting “One Health” principles into practice. Vet. Parasitol..

[72] Xhaxhiu D., Kusi I., Rapti D., Kondi E., Postoli R., Rinaldi L., Dimitrova Z.M., Visser M., Knaus M., Rehbein S. (2011). Principal intestinal parasites of dogs in Tirana, Albania. Parasitol. Res..

[73] Dado D., Izquierdo F., Vera O., Montoya A., Mateo M., Fenoy S., Galván A.L., García S., García A., Aránguez E. (2012). Detection of zoonotic intestinal parasites in public parks of Spain. Potential epidemiological role of microsporidia. Zoonoses Public Health.

[74] Otero D., Alho A.M., Nijsse R., Roelfsema J., Overgaauw P., de Carvalho L.M. (2018). Environmental contamination with *Toxocara* spp. eggs in public parks and playground sandpits of Greater Lisbon, Portugal. J. Infect. Public Health.

[75] Simonato G., Cassini R., Morelli S., Di Cesare A., La Torre F., Marcer F., Traversa D., Pietrobelli M., Frangipane di Regalbono A. (2019). Contamination of Italian parks with canine helminth eggs and health risk perception of the public. Prev. Vet. Med..

